# Selective Label-Free Detection of Imidacloprid by a Graphene Quantum Dot Fluorescent Probe

**DOI:** 10.3390/ijms26062714

**Published:** 2025-03-18

**Authors:** Yu Huang, Xiaochen Liu, Tingting Feng, Xiaohua Wang

**Affiliations:** 1 College of Traditional Chinese Medicine and Food Engineering, Shanxi University of Chinese Medicine, Jinzhong 030619, China; 18835740331@163.com (Y.H.); liuxiaochen@stu.sxtcm.edu.cn (X.L.); 2 School of Pharmacy, Guilin Medical University, Guilin 541199, China; shengyaoxue@glmc.edu.cn

**Keywords:** biosensor, fluorescence, gold nanoparticles, graphene quantum dots, imidacloprid

## Abstract

We developed a fluorescence aptamer sensing method based on gold nanoparticles and graphene quantum dots for the rapid detection of imidacloprid residues in Chinese herbal medicines. In the absence of imidacloprid, gold nanoparticles are dispersed in the solution and effectively quench the fluorescence intensity of the quantum dots due to the protective effect of the aptamer. Because of the aptamer’s specific recognition of imidacloprid, a complex forms between the two compounds, and the gold nanoparticles are no longer protected by the aptamer and can aggregate. Consequently, the fluorescence intensity of the graphene quantum dots remain unquenched, resulting in fluorescence recovery. Under optimal conditions, the fluorescence intensity showed a good linear relationship with the imidacloprid concentration in the range of 100–3 × 10^4^ ng/mL. The correlation coefficient was 0.9914, and the detection limit was 52.42 ng/mL. The recoveries of imidacloprid in the yam, matrine, and aloe leaf were 92.27–101.7%, and the relative standard deviation was 0.45–4.14%. This method has potential field applications for rapid quantitative analysis of imidacloprid residue.

## 1. Introduction

Imidacloprid is a highly effective and widely used insecticide that is of great im-portance in the agricultural industry [1]. This compound belongs to the nicotinic class of ultra-efficient insecticides, offering a broad spectrum, high efficiency, low toxicity, and low residue. It induces low resistance to pests and is safe for humans, livestock, plants, and natural predators. Furthermore, the efficacy of imidacloprid can be established on contact and via stomach action. However, the primary mechanism of action of imidacloprid is to interfere with the nervous system of insects [2]. When insects ingest imidacloprid, it blocks the transmission of impulses, causing failure of normal function and eventually death [3]. This mode of action renders imidacloprid an effective insecticide against a variety of pests, such as aphids, planthoppers, whiteflies, leafhoppers, and thrips [4]. Imidacloprid is highly useful in the production of traditional Chinese medicine (TCM) decoctions in terms of controlling pests to reduce their harm on crops and herbs [5] and ensuring a higher production yield of plants used in TCM. However, the excessive use of imidacloprid can harm the human body and pollute the environment. Therefore, a practical method for the accurate detection of pesticide residues in TCM decoctions must be developed.

The methods for the detection of imidacloprid include high-performance liquid chromatograph [6], gas chromatography [7], and immunoassay [8]. High-performance liquid chromatography is simple to perform but requires specialized equipment. Gas chromatography yields fast results but requires the sample to be processed. Immunoassay is highly sensitive but expensive. An aptamer is a type of nucleic acid molecule that can bind to a specific target with high specificity and affinity [9] through in vitro screening and evolution. The high selectivity and good stability, specificity, and sensitivity of molecular recognition using aptamers have also attracted much attention [10]. Aptamers can be used not only to detect pesticide residues but also as an innovative pesticide delivery system to enhance the efficiency of pesticide utilization and reduce environmental pollution [11,12].

Biosensors have developed rapidly in recent years because of their advantages of speed, mobility, and high sensitivity [13]. Among them, nanomaterials are widely used in the construction of sensors because of their unique characteristics. Gold nanoparticles (AuNPs) are widely used in aptamer biosensing because of their excellent biocompatibility, biostability, and unique optical properties [14,15]. Graphene quantum dots (GQDs) are a special type of carbon nanomaterial that has numerous applications in fluorescence sensing because of its excellent fluorescence properties, stability, safety, and environmental protection [16].

**Figure 1:**
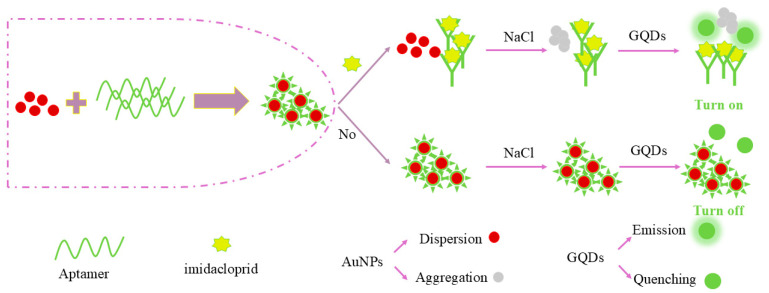
Schematic diagram of the fluorescent aptamer sensor developed for the detection of imidacloprid.

In this study, AuNPs and GQDs were used to implement a simple signal-on strategy for the unlabeled fluorescence detection of imidacloprid residues in TCM decoction specimens. As shown in Figure 1, the aptamer (Apt) is first modified to protect the AuNPs and prevent their aggregation at high concentration of NaCl, thereby safeguarding the optical properties of the AuNPs and their ability to quench the fluorescence of GQDs. This is because the electrostatic repulsion generated by the citrate keeps the synthesized gold nanoclusters dispersed, preventing them from aggregating. When NaCl is added, the negative charge of citrate is neutralized, resulting in the aggregation of gold nanoparticles. When Apt is added to the AuNPs solution, aptamers can be strongly absorbed on the AuNPs surface through the coordination of nitrogen atoms with AuNPs, thus enhancing the stability of AuNPs against salt-induced aggregation. When imidacloprid is added, it binds specifically to the surrounding modified Apt to form an imidaclopride–Apt complex. After losing the protection of the Apt, AuNPs aggregate under high NaCl concentrations and cannot quench the fluorescence of GQDs, resulting in an increase in the fluorescence intensity of the solution. Based on this principle, an imidacloprid sensing method based on fluorescence aptamer sensing was developed in this study.

## 2. Results and Discussion

### 2.1. Characterization of AuNPs

**Figure 2:**
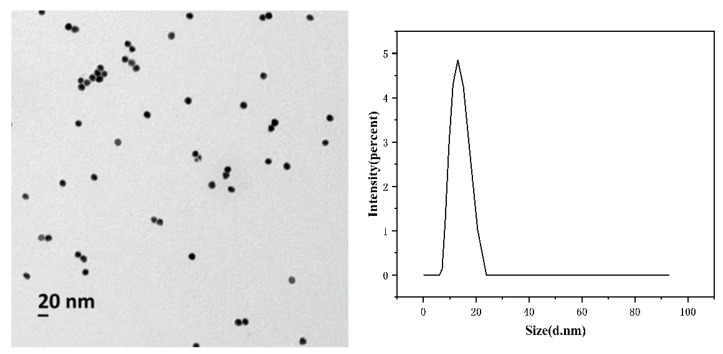
TEM characterization and DLS image of AuNPs.

As shown in Figure 2, AuNPs were characterized using transmission electron microscopy. The image shown in Figure 2 indicates that the size distribution of the prepared AuNPs was relatively uniform and well dispersed. In order to further study the size distribution and surface properties of gold nanoparticles, the particle size of AuNPs was detected by DLS measurement. It can be seen that the size of AuNPs is about 13 nm.

### 2.2. Characterization of GQDs

**Figure 3:**
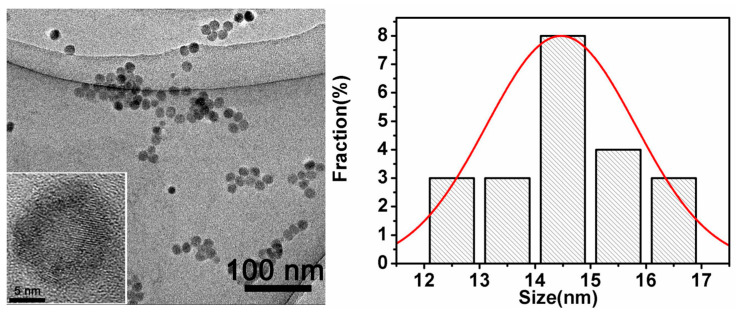
TEM images of GQDs and size distribution of aminated graphene quantum dots N-200.

The TEM images in Figure 3 show that the GQDs were spherical in morphology with an average diameter of 14.5 nm.

### 2.3. Feasibility Analysis

**Figure 4:**
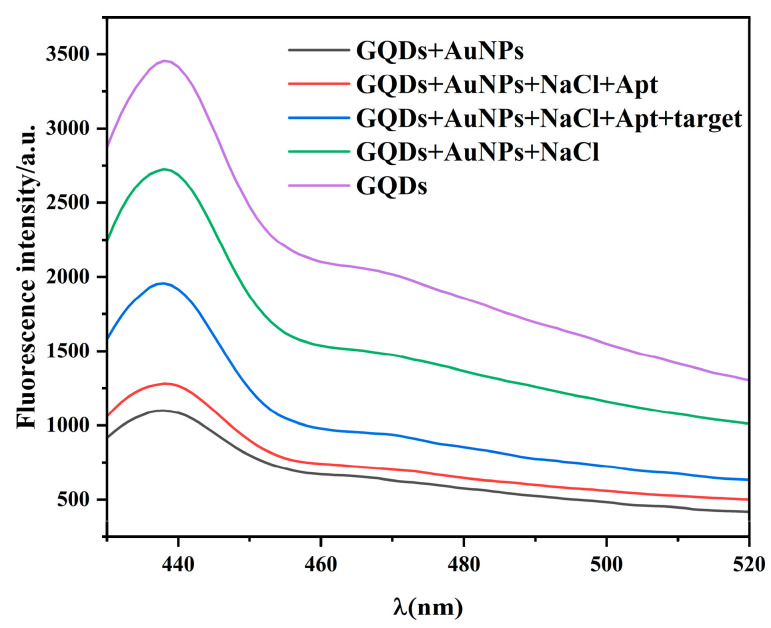
Feasibility analysis of the fluorescent sensors. Purple line: GQDs (8 μg/mL); black line: GQDs (8 μg/mL) + AuNPs (300 μL); green line: GQDs (8 μg/mL) + AuNPs (300 μL) + NaCl (8 mmol/L); red line: GQDs (8 μg/mL) + AuNPs (300 μL) + NaCl (8 mmol/L) + Apt (30 nmol/L); blue line: GQDs (8 μg/mL) + AuNPs (300 μL); NaCl (8 mmol/L) + Apt (30 nmol/L) + imidacloprid (20 μg/mL).

To verify the feasibility of the proposed method, the fluorescence spectra of the detection system in different environments were recorded (Figure 4). The fluorescence intensity was highest when only GQDs were present in the solution. With the addition of AuNPs, the FRET phenomenon occurs between the AuNPs and GQDs, and the fluorescence intensity of the AuNPs significantly decreases. When AuNPs, GQDs, and NaCl were present in the solution, the fluorescence intensity increased, indicating that the high-concentration NaCl solution induced AuNPs accumulation, decreased the FRET phenomenon, and increased the fluorescence intensity of the system. When AuNPs, GQDs, NaCl, and Apt were present in the solution, the fluorescence intensity was somewhat lower than that in the absence of Apt. This is because Apt can modify AuNPs and prevent AuNPs aggregation. FRET occurred between the AuNPs and GQDs, thereby decreasing the fluorescence intensity of the system. When AuNPs, GQDs, NaCl, Apt, and imidacloprid were present in the solution, the fluorescence intensity was higher than that in the solution without imidacloprid. This is because Apt combines with imidacloprid to form a relatively stable complex. The Apt around the modified AuNPs decreased, and more AuNPs accumulated. In turn, the FRET phenomenon decreased, and the fluorescence intensity increased. The above results demonstrate that the developed fluorescence aptamer sensing method is feasible for imidacloprid detection.

### 2.4. Optimization of Imidacloprid Detection Conditions

To achieve the best detection results, several key factors affecting the detection process were optimized, including the NaCl concentrations, the Apt concentration, and the incubation time between Apt and carbendazim.

#### 2.4.1. Effect of Aptamer Concentration on Biosensors

**Figure 5:**
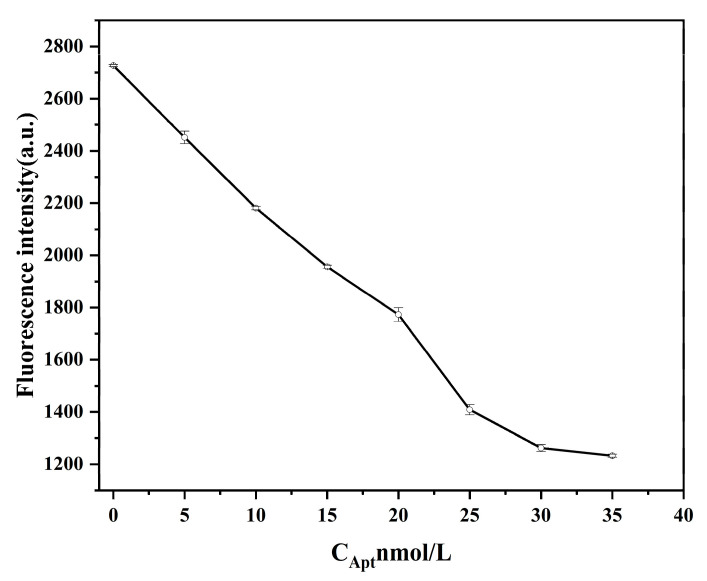
Effect of different aptamer concentrations (0, 5, 10, 15, 20, 25, 30, and 35 nmol/L) on fluorescence intensity. Other conditions: graphene carbon quantum dots (8 μg/mL); gold nanoparticles (300 μL).

The fluorescence intensity decreased with increasing Apt concentration (Figure 5). When the Apt concentration in solution reached 30 nmol/L, the fluorescence intensity of the system tended to stabilize, perhaps because AuNPs are protected sufficiently by Apt and cannot be aggregated by NaCl. Free AuNPs quench the fluorescence of GQDs. Therefore, the Apt concentration used was 30 nmol/L in subsequent experiments.

#### 2.4.2. Effect of NaCl Concentration on the Performance of Biosensors

**Figure 6:**
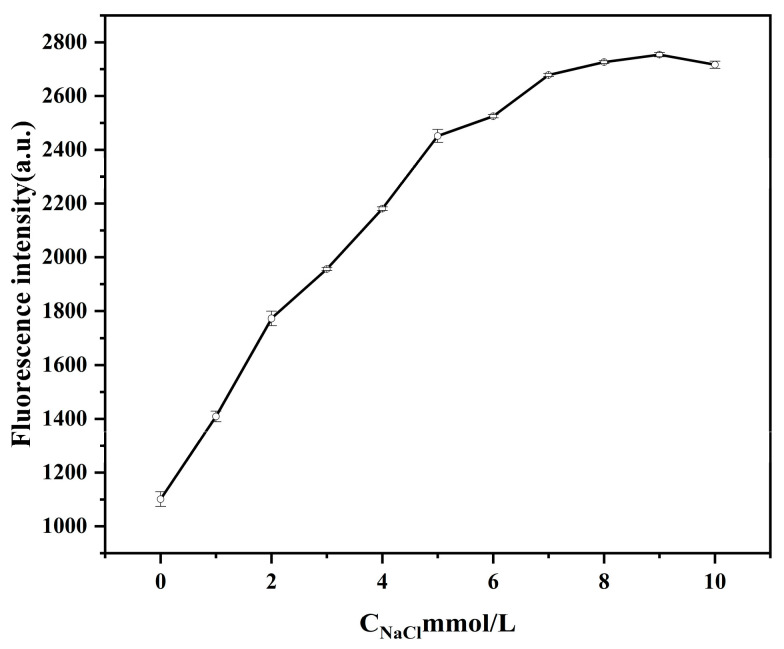
Effect of different concentrations of NaCl (0, 1, 2, 3, 4, 5, 6, 7, 8, 9, and 10 mmol/L) on the fluorescence intensity. Other conditions: graphene carbon quantum dots (8 μg/mL); gold nanoparticles (300 μL); aptamer (30 nmol/L).

As shown in Figure 6, fluorescence intensity increased with NaCl solution concentration. When the concentration of the NaCl solution reached 8 mmol/L, the fluorescence intensity tended to stabilize, indicating that all AuNPs in the system aggregated without the protection of aptamers, which could not quench the fluorescence of GQDs. Thus, the fluorescence intensity increased. Therefore, the NaCl concentration used was 8 mmol/L in the subsequent experiment.

#### 2.4.3. Study on pH of Buffer Solution

**Figure 7:**
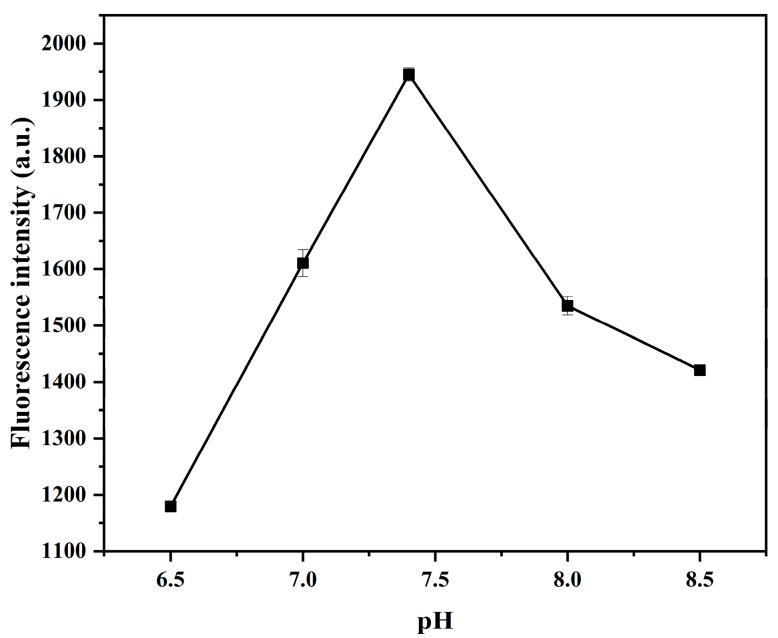
pH (6.5, 7, 7.5, 8.0, and 8.5) of the buffer solution. Other conditions: graphene quantum dots (8 μg/mL); gold nanoparticles (300 μL); aptamer (30 nmol/L); NaCl (8 mmol/L); imidacloprid (15 μg/mL).

As shown in Figure 7, when the pH of the buffer solution was 7.4, the fluorescence intensity of the system was the strongest, so the pH value of the buffer solution was 7.4 for the follow-up experiments.

#### 2.4.4. Influence of Temperature of the Sensing System

**Figure 8:**
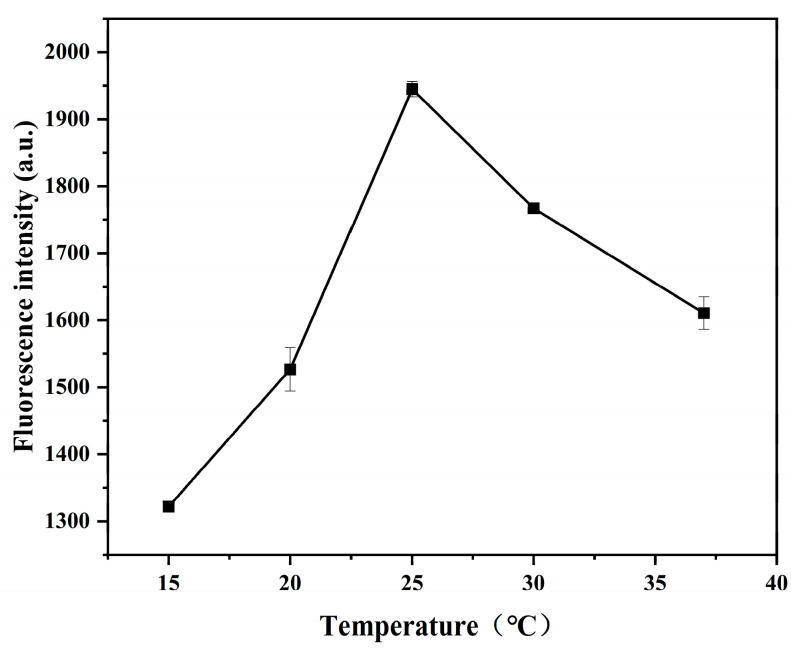
Temperature of the sensing system (15, 20, 25, 30, and 35 °C). Other conditions: graphene quantum dots (8 μg/mL); gold nanoparticles (300 μL); aptamer (30 nmol/L); NaCl (8 mmol/L); imidacloprid (15 μg/mL).

As shown in Figure 8, when the temperature of the sensing system was 25 °C, the fluorescence intensity of the system was the strongest, so the temperature was 25 °C for the follow-up experiments.

#### 2.4.5. Effect of Incubation Time of Imidacloprid and Aptamer on Fluorescence Intensity of Biosensor

**Figure 9:**
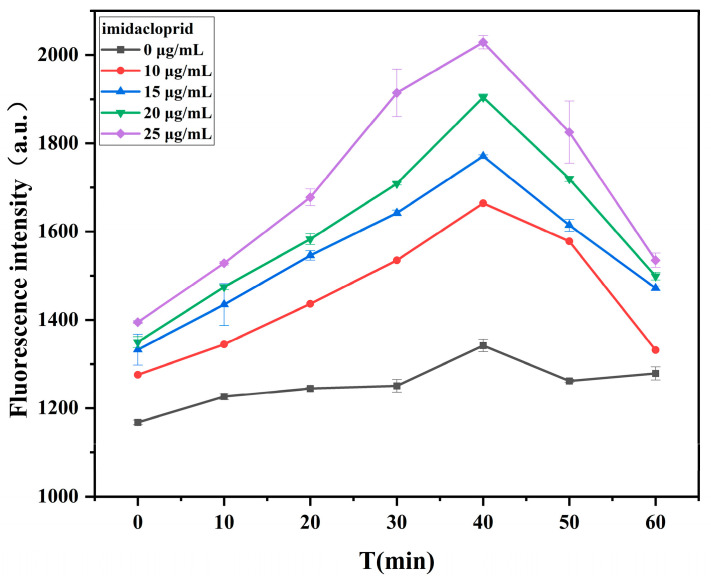
Reaction time spectra of aptamer and imidacloprid. Other conditions: graphene quantum dots (8 μg/mL); gold nanoparticles (300 μL); aptamer (30 nmol/L); NaCl (8 mmol/L).

To minimize the processing time and ensure the full combination of Apt and imidacloprid, we optimized the binding time of aptamer and imidacloprid. The fluorescence intensity of the system increased gradually with time up to and was the highest at 40 min (Figure 9). After 40 min, the fluorescence intensity of the system decreased with increasing time. Thus, the aptamer and imidacloprid were able to fuse completely at 40 min. So, it was set as the incubation time for subsequent experiments.

### 2.5. Analysis of the Detection Sensitivity of the Fluorescence Sensor

**Figure 10:**
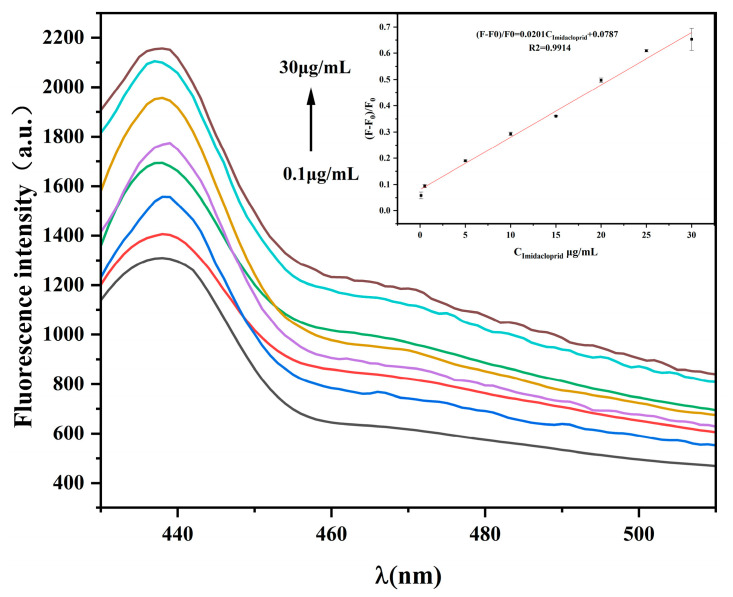
Biosensor detection of different imidacloprid concentration spectra. The lines from the bottom to the top correspond to the concentration: 0.1, 0.5, 5, 10, 15, 20, 25, 30 μg/mL. Insert: Linear curve between the fluorescence signal (F − F_0_)/F_0_ and the logarithm of different imidacloprid concentrations. Other conditions: graphene quantum dots (8 μg/mL); gold nanoparticles (300 μL); aptamer (30 nmol/L); NaCl (8 mmol/L).

To evaluate the performance of the sensor in detecting imidacloprid, the relationship between fluorescence intensity and imidacloprid concentration was investigated under the above optimal reaction conditions. Within the concentration range of 100–3 × 10^4^ ng/mL, (F − F_0_)/F_0_ was positively correlated with the imidacloprid concentration (Figure 10). The linear relationship was expressed as (F − F_0_)/F_0_ = 0.0201C_Imidacloprid_ + 0.0787, and the correlation coefficient was R^2^ = 0.9914 (where F indicates the fluorescence intensity after imidacloprid addition, and F_0_ represents the fluorescence intensity without imidacloprid). Based on the 3σ/s principle, the calculated limit of detection was 52.42 ng/mL. Compared with previously reported imidacloprid detection methods (Table 1), the present method demonstrated better detection performance. Furthermore, compared with other methods that require expensive fluorescent dye labeling and complex nanomaterial preparation, our detection process is a simpler and faster new method for imidacloprid detection.

### 2.6. Biosensor Specificity Analysis

**Figure 11:**
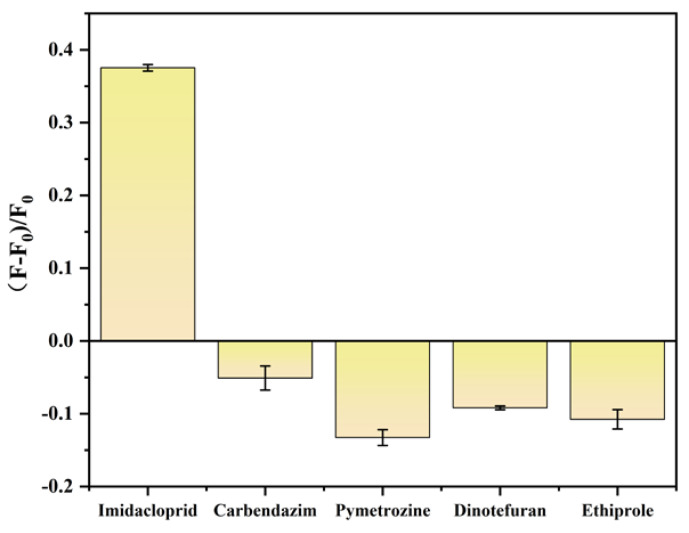
Biosensor response to different substances. Other conditions: graphene quantum dots (8 μg/mL); gold nanoparticles (300 μL); aptamer (30 nmol/L); NaCl (8 mmol/L); imidacloprid (15 μg/mL); carbendazim (150 μg/mL); pymetrozine (150 μg/mL); dinotefuran (150 μg/mL); ethiprole (150 μg/mL).

Under optimal experimental conditions, the selectivity of the fluorescent biosensor was investigated by comparing imidacloprid with four other common pesticide residue contaminants, namely carbendazim, pyrhexone, furofuran, and acetonitrile (150 μg/mL). These pesticide disruptors only produced a very low fluorescence response at the 150 μg/mL concentration, while the fluorescence intensity significantly increased in the presence of imidacloprid (15 μg/mL), indicating the high specificity for imidacloprid of our proposed biosensor (Figure 11).

### 2.7. Stability Analysis of Biosensors

**Figure 12:**
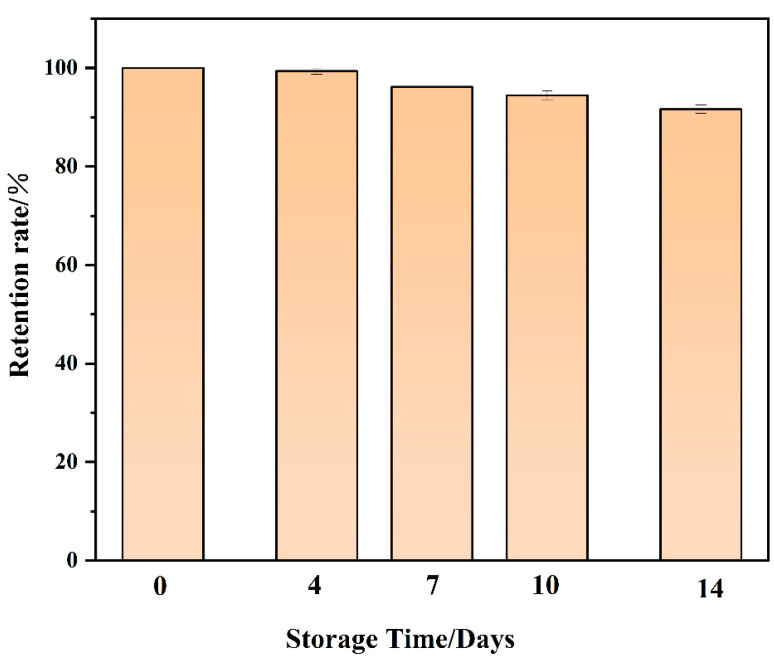
Stability of biosensors. Other conditions: graphene quantum dots (8 μg/mL); gold nanoparticles (300 μL); aptamer (30 nmol/L); NaCl (8 mmol/L); imidacloprid (15 μg/mL).

The stability of the sensor was also investigated. Three samples were prepared in parallel, and the fluorescence signal was measured after the samples were left to stand for 0, 4, 7, 10, and 14 days. In order to prevent aptamer degradation, the samples were stored at −18 °C. The biosensor was able to maintain a 91.7% signal after 14 days of placement, indicating good stability (Figure 12).

### 2.8. Testing of Chinese Medicine Samples

The accuracy and practicability of the proposed method were then verified. This method was used in the recovery experiment of Chinese herbal specimens of yam, matrine, and aloe leaf. The results are shown in Table 2. The imidacloprid recoveries in the three samples ranged from 92.27 to 101.7%, and the RSD ranged from 0.45 to 4.14%, indicating that the method exhibits good accuracy and practicability.

## 3. Materials and Methods

### 3.1. Reagents and Chemicals

The aptamer sequence of imidacloprid was 5′-TGT CGT CTA CGG TTT TGG TTG TTG TTT GTT GGT GGG TGT A-3′, which was provided by Shanghai Shenggong Biological Engineering Co., Ltd. (Shanghai, China). NaCl, auric chloride, sodium chloride, carbendazim, pymetrozine, dinotefuran, and ethiprole were purchased from Shanghai McLin Co., Ltd. (Shanghai, China). Imidacloprid was purchased from Puxi Tang Bio-technology Co., Ltd. (Tianjin, China). Yam, matrine, and aloe vera were purchased from a pharmacy. Graphene quantum dots were purchased from Nanjing Xianfeng Nano-material Technology Co., Ltd. (Nanjing, China). Phosphate-buffered saline (PBS) (10 mmol/L, pH 7.4), composed of Na_2_HPO_4_·12H_2_O, KH_2_PO_4_, NaCl, and KCl, was prepared and used in subsequent experiments.

### 3.2. Instrumentation

Fluorescence spectra were recorded using an RF-6000 fluorescence spectropho-tometer (Shimazu, Kyoto, Japan). Both excitation and emission wavelengths exhibited 5 nm bandwidths. A pHS-3E pH meter (Shanghai, China) was used to measure the pH, and a PR124ZH/E electronic balance (Changzhou, China) was used for weighing. An MTH-100 constant-temperature shaker incubator was used for the incubation baths.

### 3.3. Optimization of Biosensors

#### 3.3.1. Optimization of Apt Concentration

Apt solutions of different concentrations (0, 5, 10, 15, 20, 25, 30, and 35 nmol/L) and a 300 μL AuNPs solution were added to a 0.5 mL centrifuge tube, mixed, and incubated for 10 min. NaCl solution (8 mmol/L) was added, and the mixture was incubated for 5 min. Finally, 20 μL GQDs and PBS buffer were added, and fluorescence detection was performed after 2 min. The excitation wavelength was 380 nm, and the emission wave-length was 430 nm. All the above reactions were performed at room temperature.

#### 3.3.2. Optimization of NaCl Concentration

An Apt solution (20 μL; 30 nmol/L) and a 300 μL AuNPs solution were added into a 0.5 mL centrifuge tube and incubated for 10 min. A NaCl solution of different concentrations (0, 1, 2, 3, 4, 5, 6, 7, 8, 9, and 10 mmol/L) was added and incubated for 5 min. Finally, 20 μL GQDs and a buffer were added, and fluorescence detection was performed after 2 min. The excitation wavelength was 380 nm, and the emission wavelength was 430 nm. All the above reactions were performed at room temperature.

#### 3.3.3. Optimization of Incubation Time Between Aptamer and Imidacloprid

An Apt solution (20 μL; 30 nmol/L) and an imidacloprid standard solution (0, 10, 15, 20, and 25 μg/mL) were mixed and incubated at room temperature for 0, 10, 20, 30, 40, 50, and 60 min. An AuNPs solution (300 μL) was added, and the mixture was incubated for 10 min. A NaCl solution (8 mmol/L) was added to the mixture, which was incubated for 5 min. Finally, 20 μL GQDs and a buffer were added to the mixture, and the fluorescence was measured after 2 min at an excitation wavelength of 380 nm and emission wavelength of 430 nm. The fluorescence intensity was measured at the highest point for data analysis.

### 3.4. Evaluation of the Detection Sensitivity of the Fluorescence Sensor

An Apt solution (20 μL; 30 nmol/L) and different concentrations of an acetamidine standard solution (0.1, 0.5, 5, 10, 15, 20, 25, and 30 μg/mL) were mixed into a 0.5 mL centrifuge tube and incubated at room temperature for 40 min. An AuNPs solution (300 μL) was added, and the mixture was incubated for 10 min. A NaCl solution was added, and the mixture was incubated for another 5 min. Finally, 20 μL GQDs and a buffer were added, and the fluorescence was measured after 2 min at an excitation wavelength of 380 nm and emission wavelength of 430 nm. The highest fluorescence intensity point was used for data analysis.

### 3.5. Identification of Imidacloprid in TCM Specimens

Yam, matrine, and aloe leaf samples were selected for the sensing test. The TCM specimens were dried at 60 °C and crushed. Powdered TCM decoction specimens (1.0 g) were accurately weighed and placed in a 15 mL centrifuge tube, and 10 mL of 70% (c/c) methanol aqueous solution was added. After ultrasonic treatment for 10 min, the supernatant was collected by centrifugation at 4000 rpm for 10 min. After filtration through a 0.22 μm microporous filter membrane, the filtrate was acquired as the test solution and stored at 4 °C away from light. Then, the standard recovery experiment was performed. An Apt solution (20 μL; 30 nmol/L), an acetamidine standard solution (5, 10, and 15 μg/mL), and the test product solution were mixed into a 0.5 mL centrifuge tube and incubated at room temperature for 40 min. An AuNPs solution (300 μL) was added to the mixture, which was incubated for 10 min. A NaCl solution was added, and the mixture was incubated for 5 min. Finally, 20 μL GQDs and a buffer were added, and fluorescence was measured after 2 min. The experiment was performed in triplicate, and the recovery rate and relative standard deviation (RSD) were calculated. The excitation wavelength was 380 nm, and the emission wavelength was 430 nm.

## 4. Conclusions

In this study, a biosensor based on FRET between AuNPs and GQDs was con-structed to detect imidacloprid residues in Chinese herbal medicines. Under optimal experimental conditions, the linear range of the fluorescence signal change value (F − F_0_) F_0_ and imidacloprid concentration in the fluorescence aptamer sensing method was 100–3 × 10^4^ ng/mL, and the detection limit was 52.42 ng/mL. The method demonstrated good specificity, simple operation, low cost, and high sensitivity and required unprocessed TCM samples. The method successfully detected imidacloprid residues in the leaves of yam, matrine, and aloe vera, which are TCM herbs. In addition, the biosensor can be extended to the detection of other pesticides simply by changing the relevant aptamer on the biosensor. Finally, due to the high specificity between aptamer and imidacloprid, the biosensor has good selectivity for imidacloprid relative to other commonly used pesticides, so it is expected to be used for the rapid detection of imidacloprid in real samples.

## Figures and Tables

**Table 1:** Comparison of the different methods for imidacloprid detection.

Methods	Detection Limit (ng/mL)	Linear Range (ng/mL)	References
Imprinted polymer/reduced graphene oxide-modified glassy carbon electrode-based	51.1	1.278 × 10^2^–2.5566 × 10^5^	[17]
Electrochemical detection	66.47	1.0226 × 10^3^–1.0226 × 10^5^	[18]
Electrochemical	1.892 × 10^3^	2.3009 × 10^3^–1.2783 × 10^4^	[19]
Antifouling electro- chemical sensor	300	1 × 10^3^–4 × 10^5^	[20]
Electrochemical	2.1987 × 10^3^	7.6698 × 10^3^–5.1132 × 10^4^	[21]
The current method	52.42	100–3 × 10^4^	This work

**Table 2:** Recovery test of imidacloprid in Chinese herbal samples.

Sample	Added (μg/mL)	Found (μg/mL)	Recovery (%)	RSD (%) (*n* = 3)
Yam	5	4.66	93.13	3.77
	10	9.23	92.27	2.18
	15	14.14	94.30	0.69
Matrine	5	5.04	100.82	4.14
	10	9.93	99.36	3.93
	15	14.06	93.73	0.45
Aloe leaf	5	4.98	99.54	2.64
	10	10.17	101.7	3.02
	15	14.26	100.17	0.72

## Data Availability

The data presented in this study are available in the article.
